# Prognostic Value of Arterial Lactate Metabolic Clearance Rate in Moderate and Severe Acute Pancreatitis

**DOI:** 10.1155/2022/9233199

**Published:** 2022-11-09

**Authors:** Jiji Zeng, Jianhua Wan, Wenhua He, Yong Zhu, Hao Zeng, Pi Liu, Min Gong, Fen Liu, Qiang Shao, Liang Xia, Yin Zhu, Youxiang Chen, Nonghua Lu

**Affiliations:** ^1^Department of Gastroenterology, The First Affiliated Hospital of Nanchang University, Nanchang, China; ^2^Human Genetic Resources Center, The First Affiliated Hospital of Nanchang University, China; ^3^Department of Digestive Internal Medicine, Southern Medical University Pingxiang Hospital, Pingxiang, China; ^4^Department of Intensive Care Unit, The First Affiliated Hospital of Nanchang University, Nanchang, China

## Abstract

**Purpose:**

High lactate levels at hospital admission are significantly associated with poor prognosis in acute pancreatitis patients. Early high lactate clearance is a vital marker for predicting persistent organ failure and mortality in critical illness; however, its value in acute pancreatitis remains unclear.

**Method:**

Data were collected from patients who were diagnosed with moderately severe acute pancreatitis and severe acute pancreatitis from January 2017 to December 2020. Initial lactate (within 2 hours after admission) and repeat lactate at 24 hours after admission were measured to determine lactate clearance. Low clearance was defined as a reduction in repeat lactate of less than 30% compared to the first measurement. High clearance was defined as a repeat lactate decrease ≥30% of the first measurement or both first and second lactate levels <2 mmol/L. Baseline data, laboratory data, mortality rate, persistent organ failure rate, and other outcomes such as the incidence of septic pancreatic necrosis and sepsis and the length of hospital stay and intensive care unit (ICU) stay were compared in the low and high lactate clearance groups. Multivariate logistic regression analyses were used to assess the value of lactate clearance for predicting death.

**Result:**

Among 4425 acute pancreatitis patients, 3040 patients were diagnosed with moderate or severe acute pancreatitis, and 1028 patients had initial lactate measured. Finally, 390 patients who had initial and 24-hour repeat lactate data were included in the study. Patients who had elevated initial lactate had poor outcomes, and 51 patients in the initial elevated lactate group died. In the lactate normalization group analysis, 293 patients had 24-hour lactate normalization; compared with patients in the nonnormalization group, they had a lower rate of mortality (12.6% vs. 33%). In the lactate clearance group analysis, 70 (21.9%) patients had a low clearance after 24 hours; compared with patients in the high clearance group, they had a higher rate of developing persistent multiorgan failure (*P* = 0.045), and the incidence of death was higher (15% vs. 28.6%, *P* = 0.007). Multivariate logistic analysis showed that 24-hour lactate clearance (OR: 2.007; 95% CI:1.032-3.903, *P* = 0.04), elevated initial lactate (OR: 2.011; 95% CI:1.023-3.953, *P* = 0.043), blood urea nitrogen (OR: 2.316; 95% CI:1.061-5.056, *P* = 0.035), and white blood count (OR: 1.982; 95% CI:1.026-3.829, *P* = 0.042) were independent predictors of hospital mortality.

**Conclusion:**

The 24-hour clearance of lactate is a reliable marker to predict the outcome of moderate and severe acute pancreatitis, and low lactate clearance may indicate that the patient's condition will worsen, requiring aggressive treatments to improve patient outcomes.

## 1. Introduction

Acute pancreatitis (AP) is an acute inflammatory reaction of the pancreas; it has various causes and can lead to local complications, systemic inflammatory responses, organ failure, and even death. According to the revised Atlanta classification of 2012, AP can be classified as mild acute pancreatitis (MAP), moderately severe acute pancreatitis (MSAP), or severe acute pancreatitis (SAP) [1]. Most patients with MAP can recover in one week, but approximately 20% of patients will develop MSAP or SAP, with a mortality rate of approximately 20%-30%, and the mortality rises when persistent organ failure and/or pancreatic necrosis is present [2, 3]. The mortality of AP shows a bimodal distribution [1]. Early death is associated with persistent organ failure caused by a systemic inflammatory response, and late mortality is mainly caused by infection and the consequences of organ failure [4]. In the early stage of AP, the inflammatory cascade causes systemic inflammatory response syndrome, and tissue fluid seeps into the third space, resulting in insufficient effective circulating blood volume, microcirculatory disturbance, organ failure, and even death [3, 5, 6].

Lactate is an intermediate product in sugar metabolism, and blood lactate is usually elevated when the body experiences hypoxia or microcirculation disorders. It has also been shown that lactate concentration may be elevated in infection illnesses [7]. Lactate has been shown to serve as a potent biomarker to predict the prognosis in critical diseases, such as sepsis, trauma-related illness, and sudden cardiac arrest. Valverde-López et al. and Shu et al. showed that lactate could be a marker used to predict the severity of AP, and our previous study also showed that elevated lactate had a negative influence on acute pancreatitis [5, 8, 9]. Although lactate can be an effective biomarker to predict the outcomes of patients, lactic acid levels reflect a metabolic process in the body [10], and a single lactate value can reflect only one time point, which may not be enough to reflect changes during illness conditions. Regnier et al., Zhang at al., and Nguyen et al. showed that lactate clearance was a biomarker that can reflect treatment efficacy and disease progression over a period of time and affirmed that it was a useful marker in critically ill patients [11–13]. Therefore, our study investigated the effect of lactic acid clearance on the prognosis of patients with moderately severe and severe acute pancreatitis.

## 2. Materials and Method

### 2.1. Definition of Acute Pancreatitis

Acute pancreatitis was diagnosed according to two of three criteria: (1) typical abdominal pain, (2) elevated serum amylase and/or lipase levels up to three times the upper limit of the normal range, and (3) characteristic imaging findings of acute pancreatitis. MAP was defined as AP with no local or systemic conditions or organ failure; MSAP was defined as AP with local or systemic conditions or organ failure but not within 48 hours; SAP was defined as AP with organ failure for more than 48 hours [1]. Organ failure was defined according to the modified Marshall scoring system [1, 14].

### 2.2. Patients and Data Collection

We retrospectively collected data from consecutive patients who were diagnosed with acute pancreatitis at the First Affiliated Hospital of Nanchang University from January 2017 to December 2020. The inclusion criteria were as follows: (1) diagnosis of acute pancreatitis, with age ranging from 18 to 75 years; (2) time from abdominal pain onset to hospital no more than 72 hours; (3) initial arterial lactate tested at admission and repeat lactate tested 24 hours after admission in the hospital; (4) complete medication data. The exclusion criteria were as follows: (1) patients who had severe cardiopulmonary disease or cirrhosis; (2) pregnancy or cancer patients. Patient demographics, serum data measured within 24 hours, APACHE II scores, and outcomes of the AP patients were also collected. The study was approved by the Institutional Review Board of the hospital (No. 20223071), and all included cases were recorded in the Human Genetic Resources Center of the First Affiliated Hospital of Nanchang University.

### 2.3. Lactate Measurement and Lactate Clearance

The initial lactate measurement was obtained from the arterial blood gas analysis upon admission, and the repeat lactate measurement was obtained 24 hours after admission. Lactate <2 mmol was considered normal. 24-hour lactate was used to determine lactate clearance and normalization of lactate status. Although the indicator of lactate clearance has been studied widely, lactate acid clearance has often been assigned various values in different studies. The study by Yang CS showed that 6 hour lactate clearance ≥30.0% had a sensitivity of 60.0% in predicting survival, but Cardinal et al.'s study showed that lactate clearance ≤40% was an optimal cutoff to predict death [15]. In our study, to analyze the significance of lactate clearance in AP patients, we divided patients into a low clearance and high clearance group. Low clearance was defined as a repeat lactate reduction of less than 30% of the initial measurement. The high clearance group had repeat lactate decreases ≥30% (or both the first and second lactate levels were < 2 mmol/L). We used the following formula to calculate lactate clearance (LC): LC = (initial lactate − repeat lactate)/initial lactate × 100%.

### 2.4. Statistical Analysis

Statistical analysis was performed using IBM SPSS software, version 25.0 (SPSS, Chicago, USA). Continuous data are presented as the means and interquartile ranges (IQRs) and were analyzed by Student's *t*-test and the Mann–Whitney *U* test. Chi-square tests were used to analyze categorical variables, which are reported as numbers (frequencies). We performed multivariate logistic regression analysis to analyze the factors influencing death, and the odds ratios (ORs) and 95% confidence intervals (95% CIs) are shown. A *P*value < 0.05 was considered to indicate statistical significance.

## 3. Results

**Figure 1:**
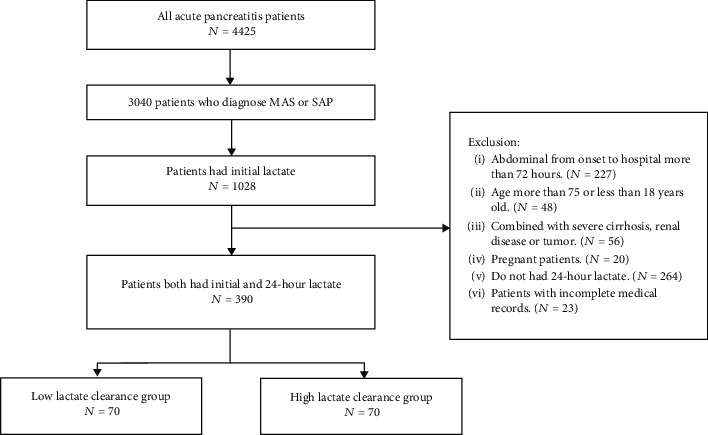
Flow chart of patient collection.

In total, 3030 patients were diagnosed with MSAP and SAP among 4425 AP patients, and only 1028 patients had an initial lactate measurement after admission. Finally, 390 patients met the inclusion criteria in our study (Figure 1). The mean age was 46 (37-59, IQR) years, and 259 (66.4%) patients were male. In our study, the main etiology was hypertriglyceridemia (44.4%). A total of 65.1% of patients had persistent respiratory failure, 96 patients developed infectious necrosis, and 17.4% had persistent multiple organ failure. The average length of hospital stay was 15 days, the average ICU stay was approximately 7 days, and the total death rate was 17.4%.

As shown in Table 1, 211 patients had an elevated initial lactate level, and 179 patients had normal lactate levels. In the elevated group, the death rate was higher than in the normal group (24.2% vs. 9.5%, *P* < 0.05), and the length of hospital and ICU stays were also higher in elevated group (*P* < 0.05). We compared the outcomes between the lactate normalization and nonnormalization groups based on the 24-hour lactate levels (Table 2). A total of 293 patients had lactate normalization after 24 hours. In the nonnormalization group, the persistent multiple organ failure rate was 35.1%, which was higher than that in the normalization group (35.1% vs. 11.6%, *P* < 0.05). Thirty-seven (12.6%) patients died in the normalization group and 31 (33%) patients died in the nonnormalization group, and the difference in hospital mortality was significant between the two groups (*P* < 0.05).

To explore the significance of lactate clearance in predicting the prognosis of AP, we compared the low clearance group and the high clearance group (Table 3). Seventy patients had a low clearance, and the mean age was 48 years old; the mean age of the high clearance group was 46 years, indicating that the two groups had no significant difference in age (*P* = 0.314). The need for mechanical-assisted ventilation in the high and low clearance groups was 32.8% and 47%, respectively, and the low clearance group was more likely to require tracheal intubation (*P* = 0.024). In the low clearance group, patients tended to develop metastasizing septicemia (*P* = 0.037). However, there was no significant difference between the groups in the length of hospital stay or ICU stay. In the high clearance group, 15.6% of patients had persistent multiorgan failure, and 25.7% of patients in the low clearance group had persistent multiorgan failure, which was a statistically significant difference. Regarding mortality, 28.6% of patients in the low clearance group died, and 15% of patients in the high clearance group died (15% vs. 28.6%, *P* = 0.007).

Table 4 provides the comparison between survivors and nonsurvivors, showing that age, white blood cell count, blood urea nitrogen, serum creatinine, PCT, APACHE II score, initial lactate level, and lactate clearance were significantly different between the two groups. However, C-reactive protein had no influence in our study. Multivariate logistic regression analysis was performed to further investigate the relationship between lactate clearance and death in AP patients. As shown in Table 5, lactate clearance was an independent predictor of in hospital mortality (OR: 2.043; 95% CI: 1.054-3.962) and blood urea nitrogen, white blood count, and initial lactate were also vital independent predictors of survival.

## 4. Discussion

Acute pancreatitis is an inflammatory condition of the pancreas that may lead to local complications, systemic inflammatory response syndrome, organ failure, and even death. In our study, AP patients with elevated initial lactate had a poor prognosis, but elevated lactate alone cannot reflect metabolization, so it may be more suitable as a marker of hazard stratification [8, 12]. Lactate clearance, calculated from two lactate values measured at different time points, can inform bedside clinicians as to whether a patient's condition is grossly improving or worsening. We investigated the association between 24-hour lactate clearance and outcomes in AP patients and showed that clearance of lactate had a vital significance in predicting multiorgan failure and death of AP patients. A low clearance may reflect that the patient's condition had worsened or that the treatment response was poor, requiring clinicians to seek more effective treatment measures.

Elevated lactate acid is always regarded to indicate hypoxia that can reflect microcirculation and has been seen as the “gold standard” [16], and lactate >2 mmol/L has a poor prognosis. The Third International Consensus Definitions for Sepsis and Septic Shock showed that elevated lactate levels are reflective of cellular dysfunction in sepsis [17]. Basic studies have shown that even if the body has no hypoxia, if mitochondrial function is damaged and the tissue cannot uptake oxygen normally, the lactic acid value will increase [18]. Obvious blood volume declines in the early period is characteristic of acute pancreatitis, hyperfusion causes organ dysfunction and reperfusion injury can exacerbate the severity of pancreatitis. Both findings make clear that microcirculation disturbance plays a vital role in the pathogenesis of acute pancreatitis [19], indicating that ameliorating microcirculation can reduce local and systemic complications [20]. In early acute pancreatitis, due to systemic inflammatory syndrome, fluid flows to the third space, leading to hypovolemia and microcirculation disorder, and insufficient tissue perfusion and hypoxia may lead to elevated lactate with acute pancreatitis. Therefore, early fluid recovery has been deemed one of the cornerstones in acute pancreatitis management [3]. Since the concept of repeating blood lactate concentration measurements was first proposed in 1983 [21], delta lactate, as a target of therapeutic protocols, has become a popular topic of research [22–25]. In recent years, lactate clearance has been used as a predictor to assess patient prognosis in many critical illnesses [26, 27]. In one prospective observational study, H. Bryant Nguyen demonstrated that a relative lactate clearance of 10% within 6 hours of initial resuscitation was an effective predictor of mortality [28]. In our study, lactate clearance ≤30% within 24 hours was associated with a high rate of multiorgan failure and death, and multivariate analysis showed that clearance ≤30% was an independent predictor of death. Although significant progress has been made in the diagnosis and treatment of acute pancreatitis in the past decade, morbidity and mortality remain high [3, 29]. Multiorgan failure has a high risk of death, and early persistent organ dysfunction and infectious pancreatic necrosis are critical factors in AP patient prognosis [30, 31]. Our study shows that 18 (25.7%) patients had persistent multiorgan failure with low clearance, which was higher than that of the high clearance group. Mizutani et al. showed that 6-hour lactate clearance after extracorporeal cardiopulmonary resuscitation was the most important predictor of in-hospital mortality in patients, and lactate clearance = 65% is a better cutoff value for extracorporeal cardiopulmonary resuscitation in predicting hospital mortality [32]. A study of severely ill children with high fever in East Africa showed that when 8-hour lactate clearance <10%, the death rate at 72-hour would increase [33]. Ha et al. showed that the median lactate clearance within 6 hours and 24 hours of survivors and nonsurvivors were different, and the clearance at 24 hours had better discriminatory power to predict hospital mortality than that at 6 hours (24-hour AUC 0.704 vs. 6-hour AUC 0.608) [34], and Masyuk et al.'s study showed that in critically ill patients, lower 24-hour lactate clearance was robustly associated with adverse outcomes [35]. Until now, there has been little standard or guided consensus clearly pointing out an accurate lactate clearance rate for predicting the death rate. However, Vincent suggested that regardless of the initial values, studying blood lactate values dynamically is necessary. The revision of the Atlanta classification suggests the time points to reevaluate lactate are 24-hour, 48-hour, and 7 days after admission to hospital, and 24 hours after admission is the right cutoff time to evaluate the treatment effect in the patient and whether the disease has progressed at the early stage [1]; however, the time will be shortened when patients are in a critical condition. In our study, we used 24-hour lactate clearance to predict the outcomes, depending on whether the clearance rate was less than 30%. Patients were divided into 2 groups, and the results showed that 24-hour lactate clearance <30% was an independent predictor of mortality. Low clearance may reflect a worsening of the patient's condition or poor treatment effects, which alerts clinicians that patients need more care or further treatment.

This study has some strengths and limitations. This is the first study to investigate the association between acute pancreatitis and arterial blood lactate clearance, to indicate that lactate clearance can be a predictor of mortality and to show that normalizing lactate in 24 hours is equally important to AP patients. However, there are also limitations. First, our study data were collected retrospectively, so selection bias was inevitable; therefore, we increased the study sample size as much as possible to minimize such bias. Hypertriglyceridemia is one of the main causes of acute pancreatitis and accounts for 1%-12% of all causes. Studies have shown that hypertriglyceridemia ranks second in some parts of China and has an upward trend in China [36, 37]. Many studies have shown that patients with hypertriglyceridemia-related acute pancreatitis develop persistent or multiple organ failure more easily, their condition is more severe, and the rate of complications is generally higher [38–40]. In our study, hypertriglyceridemia was the main cause of pancreatitis, and we excluded patients with mild pancreatitis, which may have influenced our findings. Second, we did not consider the solution type of fluid resuscitation. In an animal study, the lactate concentration increased 10 min after rapid administration of intravenous Ringer's solution (180 mL/kg/h of Ringer's solution over 60 min), but it returned to baseline values within 60 min after cessation of administration [41]. Our patients' arterial blood lactate was measured at the time of admission, and repeated blood lactate was measured after 24 hours. However, a prospective, randomized, double-blind, placebo-controlled trial indicated that intravenous Ringer's solution did not increase the blood lactate concentration [42]. Third, not every patient underwent initial or repeated arterial blood gas analysis to obtain a lactate level, and we did not monitor the WBC, BUN, PCT, CRP, and other laboratory data after 24 hours, not exploring the effect of lactate clearance, which may require us to explore further.

## 5. Conclusions

Twenty-four-hour arterial lactate clearance is a reliable marker to predict the outcome of moderate and severe acute pancreatitis, and hospital mortality increases with low lactate clearance. Lactate clearance may guide early fluid resuscitation, so continuous monitoring of lactate is necessary, especially when the condition of patients is poor. Additional prospective studies are needed to further explore the effect of lactate clearance on the prognosis of patients with acute pancreatitis.

## Figures and Tables

**Table 1:** Demographic, baseline characteristics, and outcomes of patients with moderate and severe acute pancreatitis.

	Total (*n* = 390)	Normal lactate (*n* = 179)	Elevated lactate (*n* = 211)	*P* value
Age	46 (37-59)	47 (37-60)	46 (37-59)	0.877
Male, N (%)	259 (66.4)	120 (67.0)	139 (65.9)	0.809
WBC, ×10^9^/L	13.2 (9.9-17.8)	13.0 (9-16.5)	13.6 (10.2-18.5)	0.030
Hematocrit	43.2 ± 7.8	40.8 ± 7.0	45.3 ± 8	<0.001
CRP, mg/L	212.5 (143-325.0)	204 (134-302)	216 (150-347)	0.068
Procalcitonin, ng/mL	1.4 7(0.34-5.70)	0.95 (0.31-2.42)	2.34 (0.45-10.58)	<0.001
Cr, umol/L	74 (56-113)	62 (53-83)	92 (60-155)	<0.001
BUN, mmol/L	6.3 (4.2-9.7)	5.1 (3.8-7.6)	7.3 (5.0-10.8)	<0.001
APACHE II score	10 (7-14)	9 (6-12)	11 (8-15)	0.002
SIRS score	2 (2-3)	2 (2-3)	2 (2-3)	<0.001
Mechanical assisted ventilation, N (%)	138 (35.4)	39 (21.8)	99 (46.9)	<0.001
Sepsis, N (%)	40 (10.3)	9 (5.0)	31 (14.7)	0.002
ACS, N (%)	35 (9)	5 (2.8)	30 (14.2)	<0.001
Persistent respiratory failure, N (%)	254 (65.1)	102 (57.0)	152 (72.0)	0.002
Persistent renal failure, N (%)	73 (18.7)	14 (7.8)	59 (28.0)	<0.001
Continuous circulatory failure, N (%)	10 (2.6)	2 (1.1)	8 (3.8)	0.117
PMOF, N (%)	68 (17.4)	13 (7.3)	55 (26.1)	<0.001
Infectious pancreatic necrosis, N (%)	96 (24.6)	30 (16.8)	66 (31.3)	0.001
In hospital mortality, N (%)	68 (17.4)	17 (9.5)	51 (24.2)	<0.001
Length of hospital stay, day	15 (10-26)	12 (8-18)	19 (11-33)	<0.001
ICU days, day	7 (2-14)	4 (0-9)	9 (3-19)	<0.001

WBC: white blood count; Cr: serum creatinine; BUN: blood urea nitrogen; CRP: C-reactive protein; ACS: abdominal compartment syndrome; PMOF: persistent multiple organ failure; ICU: intensive care unit; N: number.

**Table 2:** Baseline clinical characteristics and outcomes of lactate normalization and nonnormalization.

	Lactate normalization (*n* = 293)	Lactate nonnormalization (*n* = 97)	*P* value
Age, years	46 (37-60)	46 (38-59)	0.895
Male, N (%)	191 (65.2)	68 (70.1)	0.374
Etiology			0.585
Biliary, N (%)	122 (14.1)	36 (37.1)	
Alcohol, N (%)	25 (8.5)	6 (6.2)	
Hypertriglyceridemia, N (%)	127 (43.3)	46 (47.4)	
Idiopathic, N (%)	19 (6.5)	9 (9.3)	
Mechanical assisted ventilation, N (%)	88 (30)	50 (51.5)	<0.001
Sepsis, N (%)	22(7.5)	18(18.6)	<0.001
ACS, N (%)	16 (5.5)	19 (19.6)	<0.001
Persistent respiratory failure, N (%)	185 (63.1)	69 (71.1)	0.152
Persistent renal failure, N (%)	39 (13.3)	34 (35.1)	<0.001
Continuous circulatory failure, N (%)	4 (1.4)	6 (6.2)	0.009
PMOF, N(%)	34 (11.6)	34 (35.1)	<0.001
Infectious pancreatic necrosis, N (%)	66 (22.5)	30 (30.9)	0.096
Length of hospital stay, day	14 (9-23)	16 (11-33)	0.104
ICU days, day	6 (1-14)	9 (3-17)	0.030
In hospital mortality, N (%)	37 (12.6)	31 (33.0)	<0.001

ACS: abdominal compartment syndrome; PMOF: persistent multiple organ failure; ICU: intensive care unit; N: number.

**Table 3:** Clinical data compared between high- and low-clearance group.

	High-clearance group (*n* = 320)	Low-clearance group (*n* = 70)	*P* value
Age, years	46 (37-59)	48 (41-59)	0.314
WBC, ×10^9^/L	13 (10-17)	14 (10-18)	0.586
Cr, umol/L	71 (55-112)	80 (60-130)	0.086
BUN, mmol/L	6 (4-10)	7 (4-11)	0.171
ALT, U/L	28 (17-62)	34 (16-72)	0.337
AST, U/L	40 (25-72)	48 (27-104)	0.064
CRP, mg/L	217 (141-323)	203 (148-343)	0.64
PCT, ng/mL	1.5 (0.4-5.3)	1.8 (0.3-7.4)	0.606
APACHE II	10 (7-14)	11 (7-15)	0.726
Need of ventilation, N (%)	105 (32.8)	33 (47.0)	0.024
ACS, N (%)	25 (7.8)	10 (14)	0.088
Sepsis, N (%)	28 (8.7)	12 (17.1)	0.037
Persistent respiratory failure, N (%)	207 (64.7)	47 (67.1)	0.72
Persistent renal failure, N (%)	55 (17.2)	18 (25.7)	0.1
Continuous circulatory failure, N (%)	6 (1.9)	4 (5.7)	0.086
PMOF, N (%)	50 (15.6)	18 (25.7)	0.045
Infectious pancreatic necrosis, N (%)	81 (25.3)	15 (21.4)	0.486
Length of hospital stay, day	14 (9-24)	17 (12-23)	0.195
ICU length of stay, day	6 (2-14)	7 (2-14)	0.430
Hospital mortality, N (%)	48 (15)	20 (28.6)	0.007

WBC: white blood count; Cr: serum creatinine; BUN: blood urea nitrogen; ALT: alanine aminotransferase; AST: aspartate aminotransferase; CRP: C-reactive protein; PCT: procalcitonin; ACS: abdominal compartment syndrome; PMOF: persistent multiple organ failure; ICU: intensive care unit; N: number.

**Table 4:** Clinical and laboratory information of survivors and nonsurvivors.

	Survivor (*n* = 322)	Nonsurvivor (*n* = 68)	*P* value
Age, years	46 (37-58)	52 (40-64)	0.029
Male, N (%)	209 (64.9)	50 (73.5)	0.171
Etiology			0.053
Biliary, N (%)	122 (37.9)	36 (52.9)	
Hypertriglyceridemia, N (%)	153 (47.4)	20 (29.4)	
Alcohol, N (%)	25 (7.8)	6 (8.8)	
Idiopathic, N(%)	22 (6.8)	6 (27.3)	
WBC, ×10^9^/L	13 (10-17)	15 (10-23)	0.008
BUN, mmol/L	6 (4-9)	10 (6-14)	<0.001
CR, umol/L	69 (54-101)	112 (71-224)	<0.001
CRP, mg/L	211.5 (134.0-323.3)	227.0 (166.8-339.3)	0.168
PCT, ng/mL	1.1 (0.29-4.6)	4.6 (1.0-25.4)	<0.001
APACHE II score	10 (7-13)	13 (10-16)	<0.001
Initial lactate ≥ 2 mmol/L	160 (49.7)	51 (75)	<0.001
SIRS score ≥ 3	121 (37.6)	38 (55.9)	0.005
Hemoconcentration ≥ 44%	149 (46.3)	37 (54.4)	0.222
Lactate clearance < 30%, N (%)	50 (15.5)	20 (29.4)	0.007
Length of hospital days	15 (10-23)	16 (8-33)	0.574
ICU days	5 (0-11)	15 (6-27)	0.029

WBC: white blood count; BUN: blood urea nitrogen; CR: serum creatinine; CRP: C-reactive protein; PCT: procalcitonin; ICU: intensive care unit; N: number.

**Table 5:** Multivariate logistic regression analysis of risk factors for mortality.

	*P* value	OR	95% CI
Age ≥ 60 years old	0.31	1.425	0.719-2.823
White blood count ≥ 15x10^9^/L	0.042	1.982	1.026-3.829
Hemoconcentration ≥ 44%	0.403	0.765	0.408-1.433
BUN ≥ 8.9 mmol/L	0.035	2.316	1.061-5.056
Serum creatinine ≥ 133 mmol/L	0.589	1.264	0.54-2.958
CRP ≥ 150 mg/L	0.361	1.425	0.667-3.047
PCT ≥ 3.8 ng/mL	0.175	1.635	0.804-3.326
Initial lactate ≥ 2 mmol/L	0.043	2.011	1.023-3.953
Lactate clearance < 30%	0.04	2.007	1.032-3.903
SIRS score ≥ 3(1)	0.963	1.016	0.524-1.969
APACHE II score ≥ 8	0.402	1.443	0.612-3.404

BUN: blood urea nitrogen; CRP: C- reactive protein; PCT: procalcitonin; SIRS: systemic inflammatory response syndrome.

## Data Availability

The data used to support the findings of this study are available from the corresponding author upon request.

## Abbreviations

AP: Acute pancreatitis
MAP: Mild AP
MSAP: Moderately severe AP
SAP: Severe AP
LC: Lactate clearance
IQR: Interquartile range
OR: Odds ratio
ICU: Intensive care unit
PCT: Procalcitonin
AUC: Area under the curve
BUN: Blood urea nitrogen.
